# Adequate sedation with single-dose dexmedetomidine in patients undergoing transurethral resection of the prostate with spinal anaesthesia: a dose–response study by age group

**DOI:** 10.1186/1471-2253-15-17

**Published:** 2015-01-27

**Authors:** Jeongmin Kim, Won Oak Kim, Hye-Bin Kim, Hae Keum Kil

**Affiliations:** Department of Anesthesiology and Pain Medicine, Severance Hospital, Anesthesia and Pain Research Institute, Yonsei University College of Medicine, Seoul, Republic of Korea

**Keywords:** Dexmedetomidine, Aged, Sedation, Spinal anesthesia

## Abstract

**Background:**

Dexmedetomidine (DMT), a highly selective α2-adrenoceptor agonist, has been used safely as a sedative in patients under regional anesthesia. The purpose of this study was to determine the 50% effective dose (ED_50_) of single-dose DMT to induce adequate light sedation in elderly patients in comparison with younger patients undergoing transurethral resection of the prostate (TURP) with spinal anesthesia.

**Methods:**

Forty-two male patients were recruited. The young age group (Group Y) included patients 45 to 64 years old and the old age group (Group O) included patients 65 to 78 years old. After the spinal anesthesia was performed, a pre-calculated dose of DMT was administered for 10 min. The Observer’s Assessment of Alertness/Sedation (OAA/S) scale, bispectral index score (BIS) were assessed then at 2-min intervals for 20 min. A modified Dixon’s up-and-down method was used to determine the ED_50_ of the drug for light sedation (OAA/S score 3/4). In the recovery room, regression times of the motor and sensory blocks were recorded.

**Results:**

The ED_50_ of DMT was 0.25 (95% C.I. 0.15-0.35) μg/kg in Group O and 0.35 (95% C.I. 0.35-0.45) μg/kg in Group Y (*p* = 0.002). The ED_95_ was 33% lower in Group O compare with Group Y (0.38 (95% C.I. 0.29-0.39) μg/kg vs. 0.57 (95% C.I. 0.49-0.59) μg/kg). The regression time of sensory block was longer in Group O than in Group Y (109.0 ± 40.2 min vs. 80.0 ± 31.6 min) (*p* = 0.014).

**Conclusion:**

The single-dose of DMT for light sedation was lower by 21% in Group O compare with Group Y underwent TURP with spinal anesthesia.

**Trial registration:**

ClinicalTrials.gov identifier: NCT01665586. Registered July 31, 2012.

## Background

Although spinal block is the most common anesthetic method for transurethral resection of the prostate (TURP), patients may still experience adverse effects during surgery. Satisfactory sedation relieves a patient’s psychological and physiological stress and increases the patient’s acceptance of regional anesthesia. However, excessive sedation can cause adverse cardiorespiratory effects, masking the early signs of TURP syndrome, [1, 2] and even increase the incidence of postoperative delirium in elderly patients [3, 4]. Our challenge was to induce appropriately light sedation during TURP with spinal anesthesia, while maintaining the patient’s arousability, cooperativity, and cardiopulmonary stability.

Dexmedetomidine (DMT), a potent and highly selective α2-adrenoceptor agonist, has been safely used to sedate patients under regional anesthesia [5–7]. DMT induces potent sedation through its action on the locus coeruleus, the predominant brainstem nucleus involved in sleep regulation and respiratory control [8]. Compared to traditional sedatives such as midazolam and propofol, patients treated with DMT have better arousability and cooperation, minimal respiratory depression, [9, 10] and better postoperative cognitive function. Moreover, intravenous DMT hastens the onset and prolongs the duration of spinal block [11–13]. Thus, DMT could be a useful alternative for sedation in patients undergoing TURP with low-dose spinal anesthesia. DMT is usually given initially as a bolus, followed by continuous infusion. Single-dose DMT can also provide adequate sedation during short procedures under spinal anesthesia [7, 11–15]. However, previous literature investigating the single-dose use of DMT for sedation during spinal anesthesia in elderly patients is scarce [7, 11].

The aim of this study was to determine the 50% effective dose (ED_50_) and 95% effective dose (ED_95_) of single-dose DMT to induce adequate sedation in elderly patients compared to those in younger patients undergoing TURP with spinal anesthesia.

## Methods

### Clinical setting

The present study was performed at Severance Hospital from July 2012 to December 2013. This study was conducted in accordance with principles of Good Clinical Practice and was approved by the Institutional Research Board of Severance Hospital (reference number 4-2011-0927) and all patients gave written informed consent. The study was registered at ClinicalTrails.Gov with the number NCT01665586. The patients were fully informed the sedation assessment during spinal anesthesia. Exclusion criteria included the infection on puncture sites, coagulopathy disorders, allergy to local anesthetics, psychiatric history, and neurological diseases. Forty-two patients of ASA physical status I or II and who were scheduled to undergo elective TURP were recruited. Subjects were divided into two groups by the patient’s age. Patients in the young age group (Group Y) were between 45 and 64 years old and those in the old age group (Group O) were 65 to 78 years old. One anesthesiologist prepared the study drug (DMT) in a preparation room. All patients and the observational investigators were blinded to the administered dose of DMT. One millilitre (100 μg) of DMT (Precedex®, Hospira Inc., Lake Forest, USA) was diluted with 49 ml of normal saline (2 μg/ml) in a 50 ml syringe.

### Spinal anesthesia

No premedication was given. Standard monitors and bispectral index (BIS) monitors (BIS vista monitor Revision 3.0, Aspect Medical Systems, Norwood, MA, USA) were applied in the operating room. Prior to spinal block, 300 ml of lactated Ringer’s solution was administrated intravenously. Spinal puncture was performed with a 25 gauge Quincke needle at L3/4 in lateral decubitus position. After confirmation of free-flow and clear cerebrospinal fluid, 6 mg of hyperbaric bupivacaine (Marcaine® Spinal Heavy: Astra, Sodertalje, Sweden) was administered intrathecally over 10–15 s. The pin-prick test was performed then every 2 min at the mid-thoracic line bilaterally. The peak block level was defined as the same block level that persisted at four consecutive pin-prick tests. When the peak sensory block was determined, the degree of motor block was assessed using a modified Bromage Scale (in which 0 = no paralysis; 1 = unable to raise extended leg; 2 = unable to flex knee; 3 = unable to flex ankle) [16]. After the lithotomy position was done for surgical preparation, the pre-calculated amount of DMT solution was infused for 10 min using an infusion pump (Orchestra® Module DPS, Fresenius Vial S. A. S. Le Grand Chemin 38590 Brézins, France). A modified Dixon’s up-and-down method was used to determine the ED_50_ of DMT to obtain a light sedation level of an Observer’s Assessment of Alertness/Sedation scale (OAA/S) 4/3 [17]. After the infusion was completed, a second anesthesiologist evaluated the OAA/S scales, BIS scores, and vital signs every 2-min intervals for 20 min. The initial DMT dose in the first patient was 0.6 μg/kg. If the patient had a lethargic response when his name was called in a normal tone or responds only after name is spoken loudly or repeatedly (OAA/S scale 4/3) at any time point of assessment, it was defined as a successful sedation (Table 1) [18]. If the patient showed the OAA/S scale ≤2, he was awakened with tactile stimulation. If targeted sedation (OAA/S <5) was not obtained within 20 minutes, it was defined as failed sedation. Depending on the responses, the subsequent dose of DMT was decreased or increased by 0.1 μg/kg for the next patient in a stepwise fashion. Recruitment continued until six independent pairs (from successful sedation to failed sedation) would give a reliable estimation of the adequate light sedation dose of DMT in each group. Mean arterial pressure (MAP), heart rate (HR), and SpO_2_ were recorded every 5 minutes during anesthesia, at the end of surgery, at arrival and 30 min of the post anesthetic care unit (PACU).

**Table 1:** **Observer’s assessment of alertness/sedation scale** [17]

1	Does not respond to mild prodding or shaking
2	Responds to mild prodding or shaking
3	Responds only after name is spoken loudly or repeatedly
4	Lethargic response to name spoken in normal tone
5	Responds readily to name spoken in normal tone

Hypotension was defined as MAP < 60 mmHg or a > 30% decrease from the baseline value. Bradycardia was defined as heart rate < 45 bpm. Hypotension or bradycardia was treated with intravenous ephedrine or atropine. When the SpO_2_ was <92%, supplemental oxygen was given via face mask.

In the PACU, the times to sensory regression of 2-dermatomes and to motor recovery to a Bromage scale of 0 were evaluated. Postoperative pain was assessed using a verbal numerical rating scale (vNRS: 0 = no pain; 10 = worst possible pain) at 6 and 24 h postoperatively. Pain ≥ 5 vNRS was treated with 50 mg of intravenous tramadol. The time to the first analgesic request was recorded.

### Statistical analysis

Statistical analysis was performed using PASW Statistics 19™ (SPSS Inc., Chicago, IL, U.S.A.). Data was analysed by isotonic regression using the pooled-adjacent-violators algorithm (PAVA) to interpolate ED_50_ (83% C.I.) and ED_95_ (95% C.I.). Independent t-tests, chi-square tests, Fisher’s exact tests, or Mann–Whitney tests were used to compare complication rates, and postoperative pain scores between the two groups. A linear mixed model was used to analyse the hemodynamic variables across the two groups. In each group, MAP and HR changes were compared to baseline values using t-tests. The correlation of the BIS and the OAA/S scale was analyzed with a mixed model in each group. Values are expressed as mean ± standard deviation (SD), mean (95% C.I.), or as numbers. Each investigation of two groups was carried out by Dixon’s up-and-down method. The sample size was based on Dixon’s method, which requires at least six pairs of failure-success to calculate half maximal effective concentration (EC_50_) [19]. Patients were recruited until six pairs of consecutive up and down (success and failure) adjustment of the DMT dose was achieved. Statistical significance was defined by a *P* value < 0.05.

## Results

**Figure 1:**
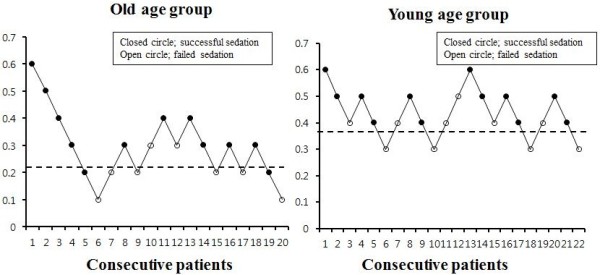
**Dixon’s up-and-down method; success (closed circle), failure (open circle).**

The sequences of successful and failed sedation are presented in Figure 1. The estimated ED_50_ of DMT was significantly different between the old and young groups [0.25 (95% C.I. 0.15-0.35) μg /kg *vs.* 0.35 (95% C.I. 0.35-0.45) μg/kg, *p* < 0.05]. When the values were interpolated using isotonic regression with PAVA and the bootstrap method, the ED_50_ (83% C.I.) and ED_95_ (95% C.I.) of DMT were still significantly different between Group O and Group Y (Table 2).

**Table 2:** **Dixon’s method and isotonic regression**

Drug dose (μg/kg)	Group Y (***n*** = 22)	Group O (***n*** = 20)
Dixon’s method		
ED_50_ (95% C.I.)	0.35 (0.35-0.45)	0.25 (0.15-0.35)
Isotonic regression method		
ED_50_ (83% C.I.)†	0.41 (0.38-0.45)	0.24 (0.19-0.3)
ED_95_ (95% C.I.)‡	0.57 (0.49-0.59)	0.38 (0.29-0.39)

ED_50_; 50% effective dose, ED_95_; 95% effective dose, C.I.; confidence interval Dixon’s method *p* value (non-parametric): 0.009.

†Isotonic regression method 83% Confidence interval: do not overlap.

‡Isotonic regression method 95% Confidence interval: do not overlap.

**Figure 2:**
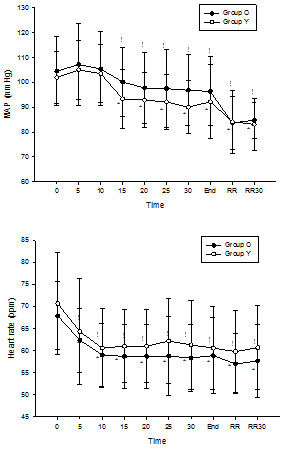
**Intraoperative hemodynamic changes.**

In both groups, MAP gradually decreased after the DMT infusion, but heart rate decreased continuously during the experiment compared to the baseline values (*p <* 0.05) (Figure 2). By linear mixed model analysis, the MAP of Group O decreased significantly more than that of Group Y (*p* < 0.05), but heart rate was not significantly different between the two groups (*p* > 0.05).

**Figure 3:**
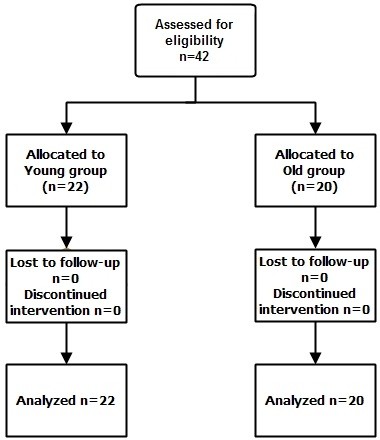
**Consort diagram.**

Forty-two patients were enrolled (Group Y = 22, Group O = 20) (Figure 3). Mean age of Group Y was 58 years old, whereas that of Group O was 72 years old. Patients’ height, weight and BSA were similar between the groups (each *p* > 0.05). The duration of surgery, maximum sensory block level and baseline hemodynamics were not significantly different between the groups (each *p* > 0.05). Comorbidities (including hypertension, diabetes mellitus, coronary artery disease and other diseases) were present in 85% of patients in Group O and 41% of patients in Group Y. The sensory block regression time was longer in Group O than in Group Y (109.0 ± 40.2 min *vs.* 80.0 ± 31.6 min, *p <* 0.05), but motor recovery time was similar in both groups. Postoperative 6 hours pain score was low in Group O compare with the Group Y (2.1 ± 0.8 *vs.* 3.2 ± 2.0, *p <* 0.05). But no difference showed in postoperative 24 hrs (Group O *vs.* Group Y, 1.4 ± 0.8 *vs.* 1.9 ± 1.5, *p >* 0.05). Transient hypotension occurred after DMT infusion in 27.3% of Group O (6/22) and 35% in Group Y (7/20). These hypotensive events were self-limited and ephedrine was not administered. In two patients of Group Y, the SpO_2_ decreased below 92% during DMT infusion but was improved with O_2_ administration. No one showed OAA/S ≤2 during the procedure. BIS and OAA/S were moderately associated in the Group O and strongly associated in the Group Y during sedation. (r = 0.489, *p <* 0.05 *vs.* r = 0.604, *p <* 0.05).

## Discussions

The first question in this study sought to determine the 50% effective dose (ED_50_) and 95% effective dose (ED_95_) of single-dose DMT to induce adequate sedation in elderly patients undergoing TURP with spinal anesthesia. The current study found that the ED_95_ of a single-dose DMT to induce light sedation was 0.38 μg/kg (95% C.I. 0.29-0.39) in elderly patients over 65 years old and 0.57 μg/kg (95% C.I. 0.49-0.59) in patients between 45 and 64 years old. The most important clinically relevant finding was the ED_95_ of single-dose DMT in elderly patients was 67% that of younger patients.

Manufacturer suggests that sedation with DMT can be achieved with 0.2-0.7 μg/kg/hr after 1.0 μg/kg loading dosage [20]. However, in elderly patients, excessive sedation can easily occur when recommend dosage of DMT was used. Park *et al*. found that excessive sedation with a Ramsay score of 5/6 occurred in 46% of patients treated with 0.5 μg/kg DMT and 60% of patients treated with 1 μg/kg DMT in elderly patients [11]. In comparison, none of the 30- to 40-year-old patients showed excessive sedation of OAA/S ≤ 2 after a 0.5 μg /kg DMT infusion [15]. Therefore, minimal optimum dose of DMT to avoid over sedation should be determined in elderly patients. The findings of this study suggest that we should reduce the dose of DMT for elderly patients as one third of recommend dose for young patients. These results match those observed in earlier studies that suggest that the required dosage of DMT in elderly patients is half that of the recommended young adult dose [21, 22].

Although, these findings cannot be extrapolated to all patients, the present study provides additional evidence with respect to pharmacokinetics of DMT in elderly patient undergoing spinal anesthesia. Generally, elderly patients are more sensitive to sedative agents. Less medication is usually required to achieve a desired clinical effect, and drug effect is often more pronounced and prolonged than we expected. However, to date there has been little agreement on the dosage of DMT in elderly patient undergoing neuraxial blockade or peripheral blockade. Whilst this study did not confirm the pharmacokinetics of DMT, it would be helpful to set out a practical guideline of DMT for the elderly patient undergoing neuraxial blockade or peripheral blockade. If a planned regional blockade is done successfully, a relatively small single-bolus dose of DMT <0.5 μg/kg would be needed to reduce discomfort, anxiety and restless in elderly patients undergoing surgical procedure within 30 minutes.

There are several possible explanations for this result. First of all, spinal anesthesia may increase sensitivity to a sedative agent, explaining the low ED_95_ for an OAA/S sedation scale [23]. Since Tverskoy *et al*. reported that spinal block with bupivacaine decreased the hypnotic requirement of midazolam, thiopental, and propofol, [24, 25] several studies have confirmed these findings [23, 26, 27]. Especially in the early stage of spinal block, spinal anesthesia decreases afferent input and this reduces the sedative hypnotic requirement. Another possible explanation for this is that spinal analgesic spread is greater and analgesic recovery is prolonged in elderly patients as compared to younger patients [28–31]. These characteristics may be related to the different pharmacokinetic and pharmacodynamic properties of local anesthetics with advancing age.

Most studies in the field of DMT have only focussed on the sedative dose as a general intravenous anesthetic, few studies have investigated single-dose DMT use in spinal anesthesia. Three randomized controlled studies reported that single-dose DMT (0.25 μg/kg, 0.5 μg/kg, or 1 μg/kg) improved spinal anesthesia by increasing block duration, increasing postoperative analgesia and achieving patient satisfaction [7, 11, 15]. Intravenous DMT is known to prolong the anesthetic duration and improve the analgesic characteristics associated with spinal block [7, 11–14]. Although the underlying mechanism for this is still unclear, supraspinal, direct analgesic, and vasoconstricting effects are thought to be involved [9, 12]. We found that sensory block duration was longer in the old age group than the young age group (109.0 ± 40.2 min *vs.* 80.0 ± 31.6 min) despite similar peak sensory block levels. These results are consistent with those of other studies.

One unanticipated finding was that BIS and OAA/S were moderately associated in the Group O and strongly associated in the Group Y during sedation. Although, there were significant correlations between OAA/S and BIS (Group O, r = 0.489, *p <* 0.05 *vs.* Group Y, r = 0.604, *p <* 0.05), we thought this statistical correlation between BIS values and OAA/S scores may not be meaningful in this dose-response study. Several studies have demonstrated that BIS is not a sensitive tool for measuring sedation induced with spinal anesthesia [18]. Furthermore, it has age-dependent differences, [32, 33]. Accordingly, we used BIS as an adjunctive for clinical reference to the OAA/S scale. EEG-based monitors did not reliably distinguish between light and deep sedation [34]. Because, our targeted sedation depth was OAA/S 3/4, relatively light sedation, BIS value could not be appropriate in this setting. In addition, Kasuya *et al*. reported a discrepancy between OAA/S and BIS scoring when DMT was used as the sedative agent [35] and elderly patients may lose consciousness at a higher BIS score than do young adults [33]. Therefore, these results need to be interpreted with caution.

## Conclusions

In conclusion, our results suggest that a single-dose of DMT for elderly patients undergoing spinal anesthesia for TURP should be reduced to two-thirds of the dose for younger patient. However, careful hemodynamic monitoring is imperative in elderly patients administered DMT for sedation under regional anesthesia, because small doses of DMT (<0.3 μg/kg) can result in bradycardia and hypotension.

## Abbreviations

BIS: Bispectral index score
DMT: Dexmedetomidine
HR: Heart rate
MAP: Mean arterial pressure
OAA/S: Observer’s Assessment of Alertness/Sedation scale
PACU: Post anesthetic care unit
PAVA: Pooled-adjacent-violators algorithm
TURP: Transurethral resection of the prostate
vNRS: verbal numerical rating scale.
